# Predictive biomarkers for negative symptoms in schizophrenia

**DOI:** 10.1192/j.eurpsy.2021.369

**Published:** 2021-08-13

**Authors:** N. Cakici, L. De Haan, N. Van Beveren

**Affiliations:** Department Of Psychiatry And Amsterdam Neuroscience, Academic Medical Center, Amsterdam, Netherlands

**Keywords:** biomarker, Inflammation, Growth Factors, negative symptoms

## Abstract

**Introduction:**

Increasing evidence shows that impaired neuroplasticity and high inflammation play a crucial role in the pathophysiology of schizophrenia. Prospective studies demonstrated that patients with high inflammation usually have a poor treatment response and clinical practice learns that negative symptoms are challenging to treat. The predictive value of biomarkers for negative symptoms in patients with schizophrenia has sparsely been explored.

**Objectives:**

Here, we investigated whether biomarkers are associated with negative symptoms at baseline, and whether biomarkers could predict negative symptoms after six years in patients with schizophrenia.

**Methods:**

We investigated serum biomarkers in an epidemiological study on schizophrenia (N, baseline=110; N, follow-up=65). Negative symptoms were measured using the Positive and Negative Syndrome Scale. Biomarkers (N=189) were measured with a multi-analyte profiling platform and analysed using linear regression models, adjusted for site, age, gender, ethnicity, anti-inflammatory agents, smoking, cardiovascular disease and diabetes, and adjusted for multiple comparisons (q, Benjamini-Hochberg procedure).

**Results:**

In particular, decreased platelet-derived growth factor (PDGF) (responsible for proliferation of oligodendrocytes) was associated with more negative symptoms at baseline and follow-up (figure). Several other biomarkers associated with inflammation, neuroplasticity and metabolism correlated with the severity of negative symptoms cross-sectionally and/or prospectively. Figure. Biomarkers for Negative Symptoms in Schizophrenia.

**Figure:**
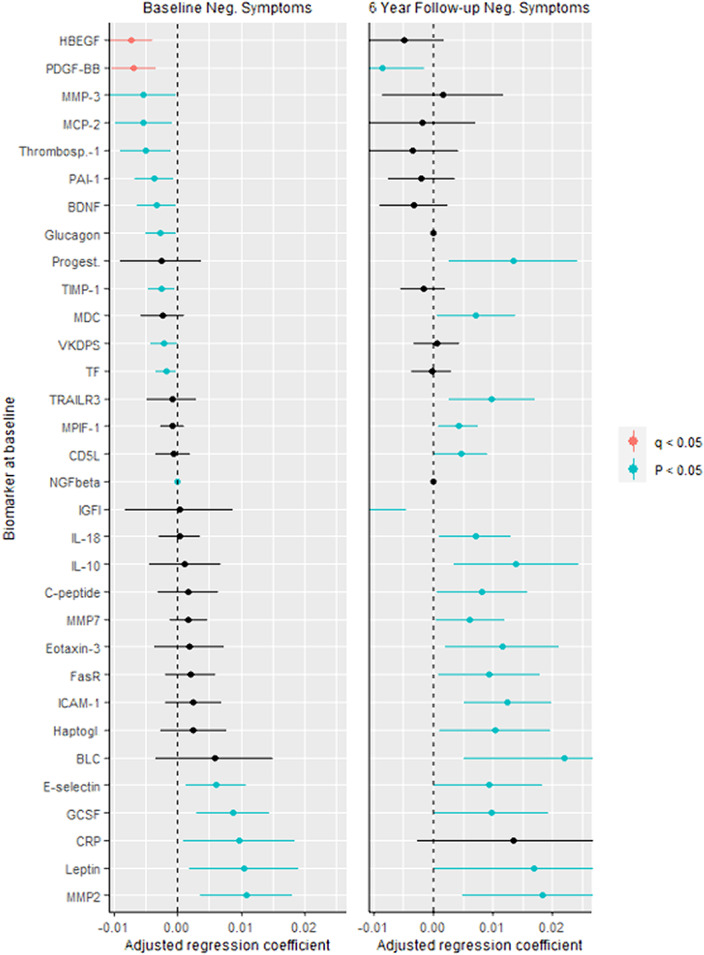

**Conclusions:**

Although our sample size at follow-up was limited, we feel that these analyses contribute to further research of possible predictive biomarkers for negative symptoms in schizophrenia. During the conference we will elaborate our findings with applied machine learning techniques which might shed more light on the predictive value of biomarkers for negative symptoms in schizophrenia.

**Disclosure:**

No significant relationships.

